# Description of a new halophilic tiger beetle in the genus *Eunota* (Coleoptera, Cicindelidae, Cicindelini) identified using morphology, phylogenetics and biogeography

**DOI:** 10.1371/journal.pone.0257108

**Published:** 2021-10-13

**Authors:** Daniel P. Duran, Stephen J. Roman

**Affiliations:** 1 Department of Environmental Science, Rowan University, Glassboro, NJ, United States of America; 2 Austin, TX, United States of America; Universita degli Studi di Roma La Sapienza, ITALY

## Abstract

Tiger beetles are a popular group of insects amongst amateur naturalists, and are well-represented in museum and private collections. New species descriptions plateaued in the 19^th^ century, but there is a recent resurgence of discoveries as integrative taxonomy methods, guided by molecular systematics, uncover “cryptic” tiger beetle diversity. In this paper, we describe a new species using multiple data types. This new species, *Eunota mecocheila* Duran and Roman **n. sp.**, is in the tribe Cicindelini, and is described from specimens collected in saline muddy ditches in northern Mexico. This species is closely related to *E*. *circumpicta* (LaFerté-Sénectère, 1841), but is separated based on morphological differences, geographic range, and genetic differentiation. Little is known about the biology or distribution of this species and it has only been collected from two sites in the state of Coahuila. Given the location of this new species, and its genetic divergence from its closest relative, *E*. *circumpicta*, we discuss the historical biogeography that may have led to isolation and speciation. The male and female dorsal, lateral and frontal habitus and the male aedeagus are shown.

## Introduction

Tiger beetles are one of the most studied groups of non-pest insects [1, 2] and have been used as models organisms in conservation biology and biodiversity assessments [3, 4]. Most of the fauna of North America had been described by the mid-1800s and by the latter half of the 1900s it appeared as if nearly all of the diversity had been discovered and named [5]. Species delineations had been based nearly exclusively on morphological characters for the vast majority of taxa, with lesser reliance on ecological or other characters. However, in recent years, taxa have been delineated and described through the integration of traditional morphology, molecular data and/or life history [5–9]. This has led to the discovery of ‘cryptic species’; that is, species that are distinct evolutionary units, but had gone undetected due to physical similarity with closely related species.

The New World tiger beetle genus *Eunota* Rivalier, 1954 includes approximately a dozen described species [10] and is distributed from the southern United States south to Brazil, reaching its highest diversity in northern Mexico. Recently, the taxonomy of this group was revised using molecular, morphological and life history data [11] and involved the transferal of nearly a dozen North American species of *Habroscelimorpha* Dokhtouroff, 1883 to the historically monotypic *Eunota*. Members of the genus are active both diurnally and nocturnally and are typically found in open or sparsely vegetated muddy or sandy saline habitats. Most species in the genus are allopatrically distributed and do not overlap with putative close relatives [12]. Two exceptions are *E*. *severa* (LaFerté-Sénectère, 1841) *and E*. *togata* (LaFerté-Sénectère, 1841), which overlap with each other as well as with coastal populations of *E*. *circumpicta*.

**Fig 1:**
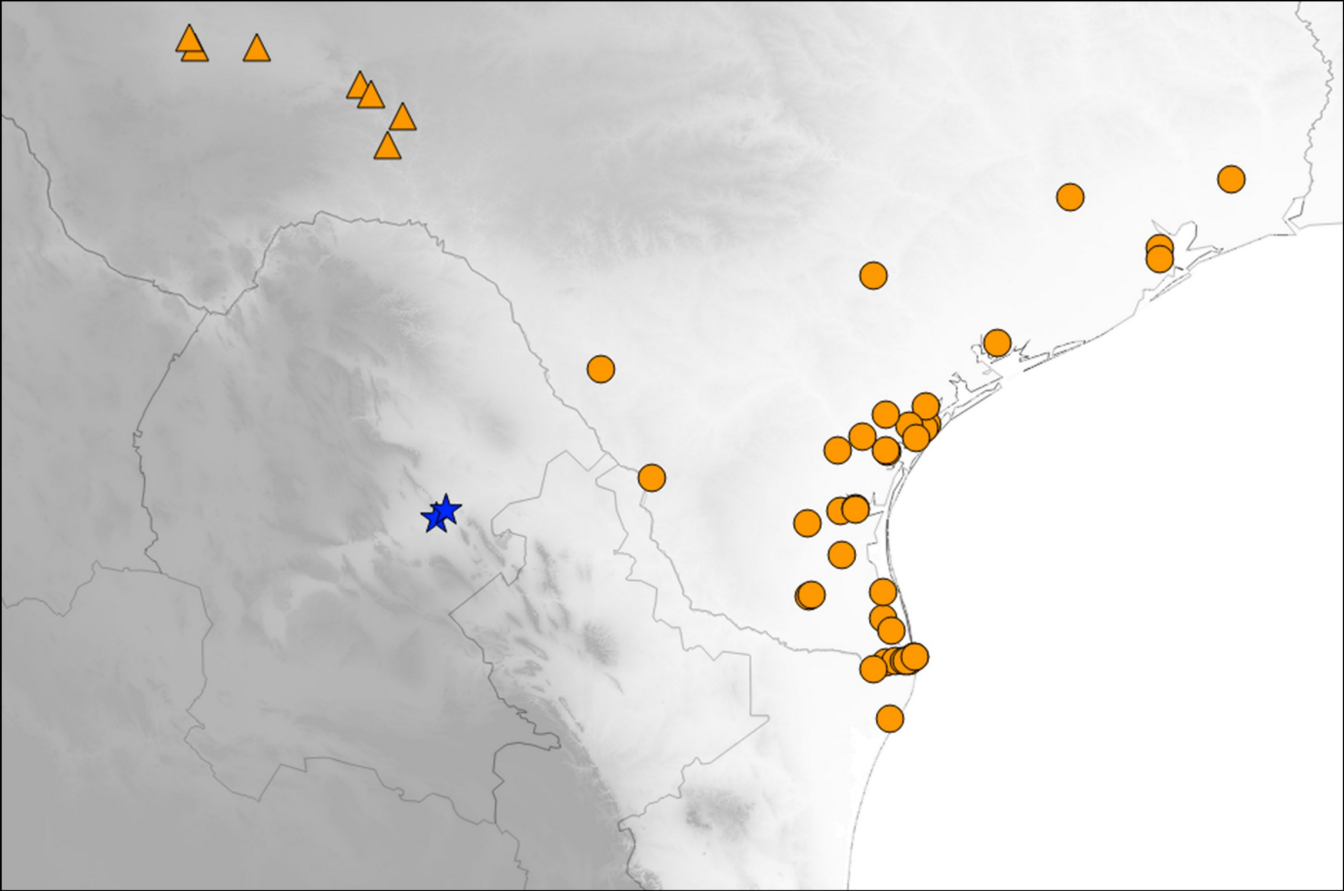
Distribution of *E*. *mecocheila* n. sp. known localities (blue stars) in Mexico. Also shown are proximate localities for *E*. *circumpicta johnsonii* (orange triangles) and *E*. *circumpicta circumpicta* (orange circles). Shading corresponds to elevation. Darker areas are at higher elevation.

Herein we describe *E*. *mecocheila* **n. sp.**, known only from saline muddy ditches in two localities within the northern Mexican state of Coahuila (Fig 1), using morphological characters and a mitochondrial DNA genealogy. Due to the species apparently isolated allopatric distribution, we discuss possible historic biogeographical scenarios that may have led to its speciation from *E*. *circumpicta*. This single species description is being published at the present time because the new taxon occupies a distinctive habitat in a restricted geographic area near to areas undergoing land development. This is a taxon of potential conservation concern.

## Materials and methods

### Ethics statement

Samples were taken along roadsides on public lands in Mexico in 1992 and 1994. The collectors had contacted Dr. Harry Brailovsky Alperowitz at the National Autonomous University of Mexico (UNAM) and he supported the collection efforts and provided written permission to local authorities in Coahuila. The holotype of *E*. *mecocheila* will be deposited in the Mexican national collection.

### Specimen collection and morphological analyses

Specimens of a putatively undescribed *Eunota* similar to *E*. *circumpicta johnsonii* were collected by Ronald L. Huber, John A. Shetterly, David Brzoska and John Stamatov during a 1992 trip to Mexico. Additional specimens were collected by the second author and Walter N. Johnson during a 1994 trip to Mexico at a second locality in Coahuila. Type material is deposited in the following institutional and private collections (acronyms used in the text are in parentheses): National Insect Collection of Mexico (CNIN), National Museum of Natural History, Smithsonian Institution, Washington, DC, USA (NMNH), Collection of Ronald L. Huber, Bloomington, MN (RLHC), Collection of Walter N. Johnson, MN (WNJC) and Collection of Stephen J. Roman (SJRC), Collection of John A. Shetterly (JASC), Collection of Daniel P. Duran (DPDC).

A total of 18 specimens were available to be examined for morphological characters. Body measurements follow Duran and Moravec [13]. The total body length excludes the labrum and is measured as the distance from the anterior margin of the clypeus to the elytral apex, including the sutural spine. The width of the pronotum is measured to include the lateral margins of the proepisterna. The width of the head is measured as the distance between the outer margins of the eyes.

Images were captured by means of a Canon EOS 1D Mark IV camera with a Canon 100mm macro lens. Images were montaged and edited with Adobe Photoshop CS6. Scale bars and measurements were calibrated with an ocular micrometer on Olympus SZ61 and SZX7 microscopes. The final digital images were processed with Adobe Photoshop CS6.

### Mitochondrial genealogical analyses

To address the placement of *E*. *mecocheila* within the genus *Eunota*, we generated an mtDNA genealogy. Although mtDNA can have limitations for phylogenetic inference due to hybridization and introgression between closely related species [9, 14, 15], this is unlikely to be a problem for *Eunota* as closely related species and subspecies are allopatric. Two specimens of the new species were sampled along with several representatives of the putative closest relative, *E*. *circumpicta*, encompassing the geographically proximate *E*. *c*. *circumpicta* and *E*. *c*. *johnsonii* subspecies. *E*. *fulgoris* and *E*. *praetextata* were also sampled as they are known to be closely related to *E*. *circumpicta* [16] and *E*. *togata* was chosen as the outgroup.

DNA extractions were performed on whole beetles in a non-destructive manner to preserve specimens for morphological examination and as voucher specimens. Extractions were performed using Qiagen DNeasy kits per the manufacturer’s protocol. A 457 bp region of the mitochondrial genome of the cytochrome b gene (*cytb*) was amplified using the CB1 and CB2 primers [17]. This gene was chosen based on its short fragment length, making it more likely to amplify for pinned specimens with degraded DNA. PCR conditions were as follows: 2 min at 96°C followed by 10 cycles of denaturation at 96°C for 30 s, annealing at 46°C for 30 s and extension at 72°C for 1 min, then followed by 30 cycles of denaturation at 96°C for 30 s, annealing at 48°C for 30 s and extension at 72°C for 1 min, with a final extension step at 72°C for 5 min. PCR products were purified using either the Geneclean II Kit (BIO 101 Inc.) or the Millipore Multiscreen 96-well plates (Millipore, Billerica), and were sequenced using BigDye chemistry and an ABI PRISM 3700 DNA Analyzer (Applied Biosystems). Fragments were sequenced using both forward and reverse primers. Sequences were first edited manually, aligned automatically and revised by eye using Geneious Prime 2021 (https://www.geneious.com). For all individuals used in analyses, sequences for the entire 457 bp fragment were complete. Sequences were deposited in the NCBI GenBank Database under the accession numbers MZ404132—MZ404270.

We inferred phylogenetic relationships with IQ-TREE v. 2.0 [18]. Partitioning was based on codon position and model selection was performed using ModelFinder in IQ-TREE by specifying the command -MFP. With this command IQ-TREE selects from over a hundred substitution models and chooses the best model using the Bayesian Information Criterion (BIC) [19]. We then conducted 500 independent tree searches and the tree with the best maximum likelihood score was selected. For each of the 500 searches, we estimated nodal support using 1000 ultrafast bootstraps (UFBoot) and 1000 SH-aLRT tests. We used the -bnni command to avoid severe model violation resulting in overestimation of nodal support when preforming ultrafast bootstraps. Pairwise genetic distances between sequences were calculated using DnaSP 6.12 [20].

### Nomenclatural acts

The electronic edition of this article conforms to the requirements of the amended International Code of Zoological Nomenclature, and hence the new names contained herein are available under that Code from the electronic edition of this article. This published work and the nomenclatural acts it contains have been registered in ZooBank, the online registration system for the ICZN. The ZooBank LSIDs (Life Science Identifiers) can be resolved and the associated information viewed through any standard web browser by appending the LSID to the prefix “http://zoobank.org/”. The LSID for this publication is:

urn:lsid:zoobank.org:pub:2BA8FFB2-A3E2-463A-B9F3-C2FE64AE3E80. The electronic edition of this work was published in a journal with an ISSN, and has been archived and is available from the following digital repositories: LOCKSS.

## Results

*Eunota mecocheila* **n. sp.** possesses a longer labrum than *E*. *circumpicta*. Labral length to width ratio for *E*. *mecocheila* is 0.55–0.7 for males, 0.55–0.65 for females; for *E*. *circumpicta* it is 0.35–0.50 for males, 0.35–0.45 for females. Morphological measurements are listed in S1 Table. *Eunota mecocheila* **n. sp.** exhibits maculation patterns and color variation that are most similar to populations of *E*. *c*. *johnsonii*, but *E*. *mecocheila* is smaller at 10.2–12.0 mm total length, mean length 11.0 mm, compared to 12.3–15.7 mm, mean length 13.1 mm for *E*. *c*. *johnsonii*. In addition, the only known populations for *E*. *mecocheila* **n. sp.** are separated by more than 350 km from the nearest population of *E*. *c*. *johnsonii*.

**Fig 2:**
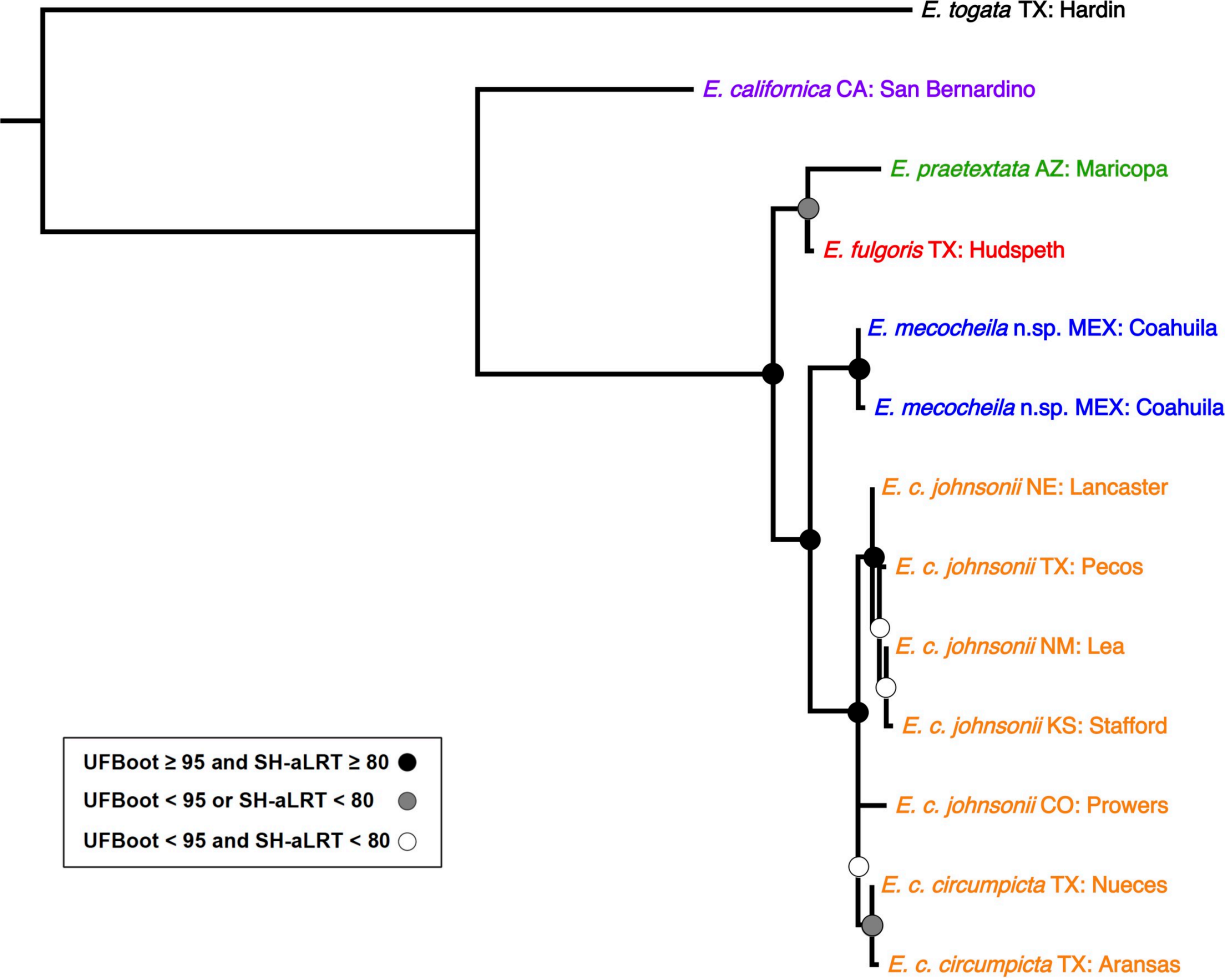
Maximum-likelihood mtDNA genealogy based on 457 bp of cytochrome b. Phylogeny inferred in IQ-TREE v.1.6.9 [18]. The root of the tree was placed between *E*. *togata* and the rest of the taxa based on Gough et al. [16]. Shown are the species names for selected *Eunota* taxa. For *E*. *circumpicta*, subspecies are indicated as well. Taxon names are followed by locality information. First are standard two-letter abbreviations for US states, with the exception of “MEX” for the county of Mexico. These are followed by counties, with the exception of “Coahuila” for the northern Mexican state.

The phylogenetic analysis recovered *E*. *mecocheila* **n. sp.** as sister clade to a monophyletic *E*. *circumpicta* (Fig 2). The clade containing *E*. *mecocheila* + *E*. *circumpicta* was sister to *E*. *fulgoris*. Statistical support for clades was high, with most nodes exhibiting UFBoot values above 95% and SH-aLRT values above 80%. *E*. *mecocheila* sequences were on average 3.3% divergent from *E*. *circumpicta*.

### Taxonomy

#### *Eunota mecocheila* Duran & Roman, n. sp

urn:lsid:zoobank.org:act:58A8C88F-5CDC-449F-9BB7-27EA820256BA

**Fig 3:**
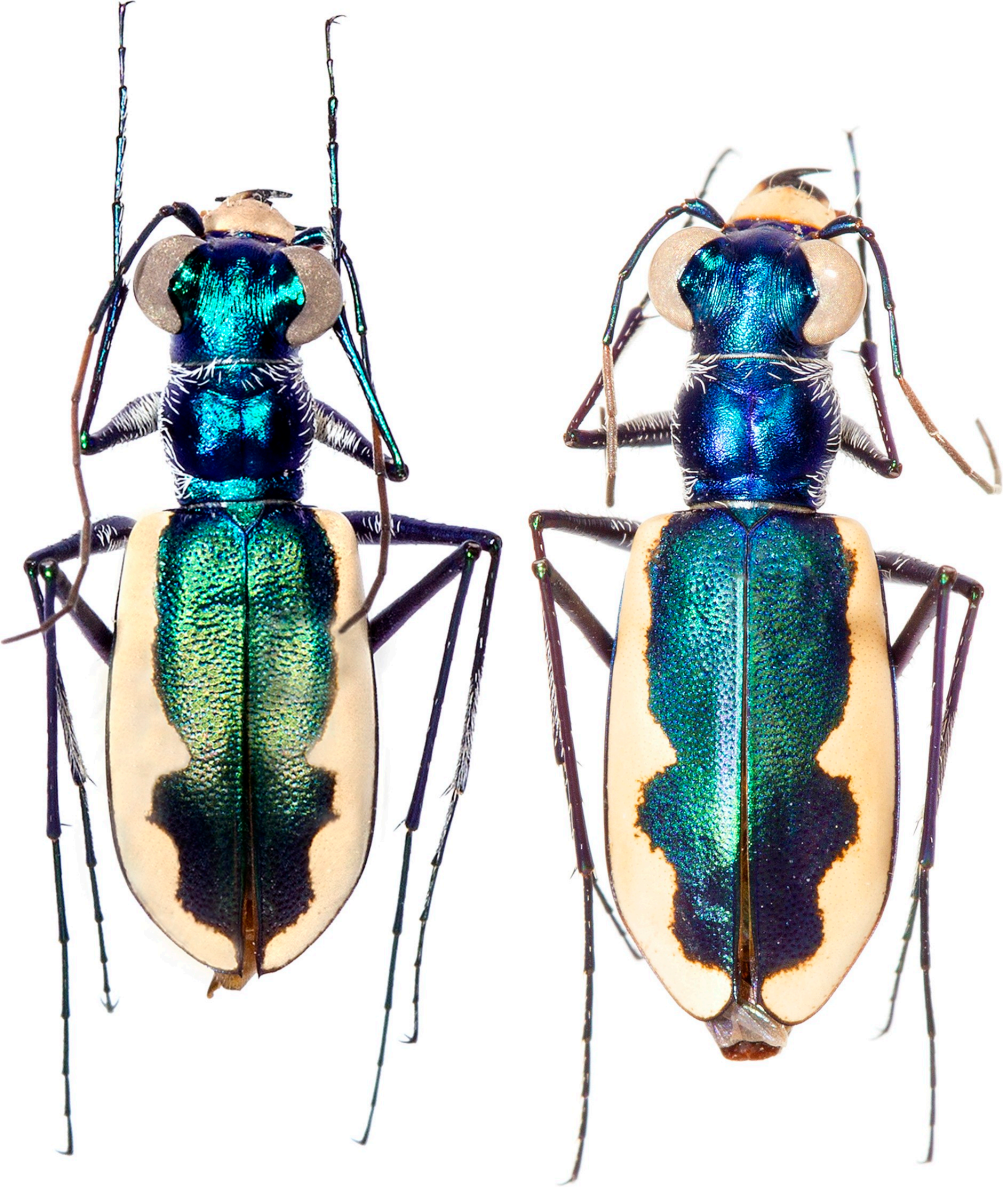
Dorsal habitus of *E*. *mecocheila* n. sp. holotype (male) and a paratype (female), respectively.

**Fig 4:**
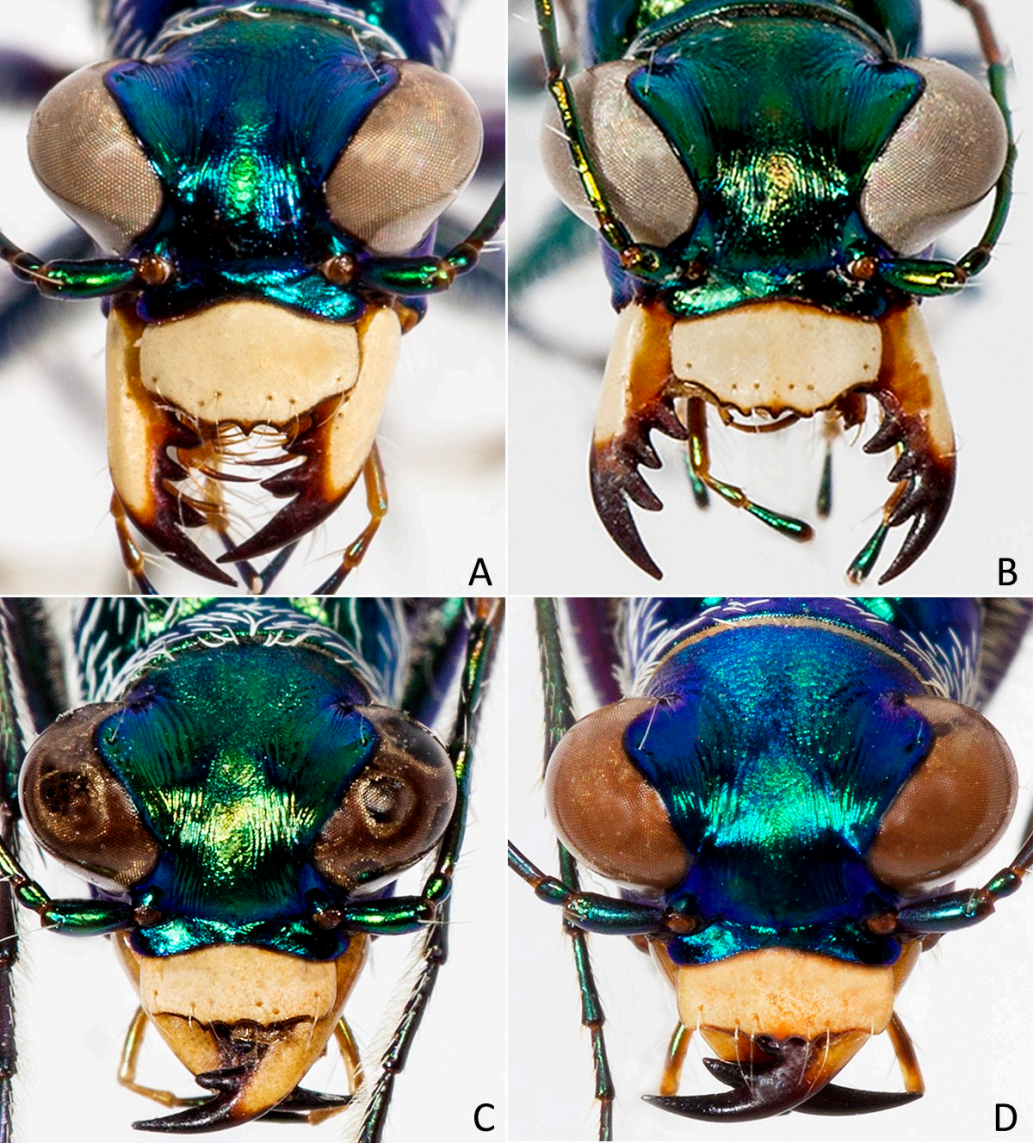
Frontal habitus A) *E*. *mecocheila* n. sp. male, B) *E*. *mecocheila* n. sp. female, C) *E*. *circumpicta johnsonii* male, D) *E*. *circumpicta johnsonii* female.

**Fig 5:**
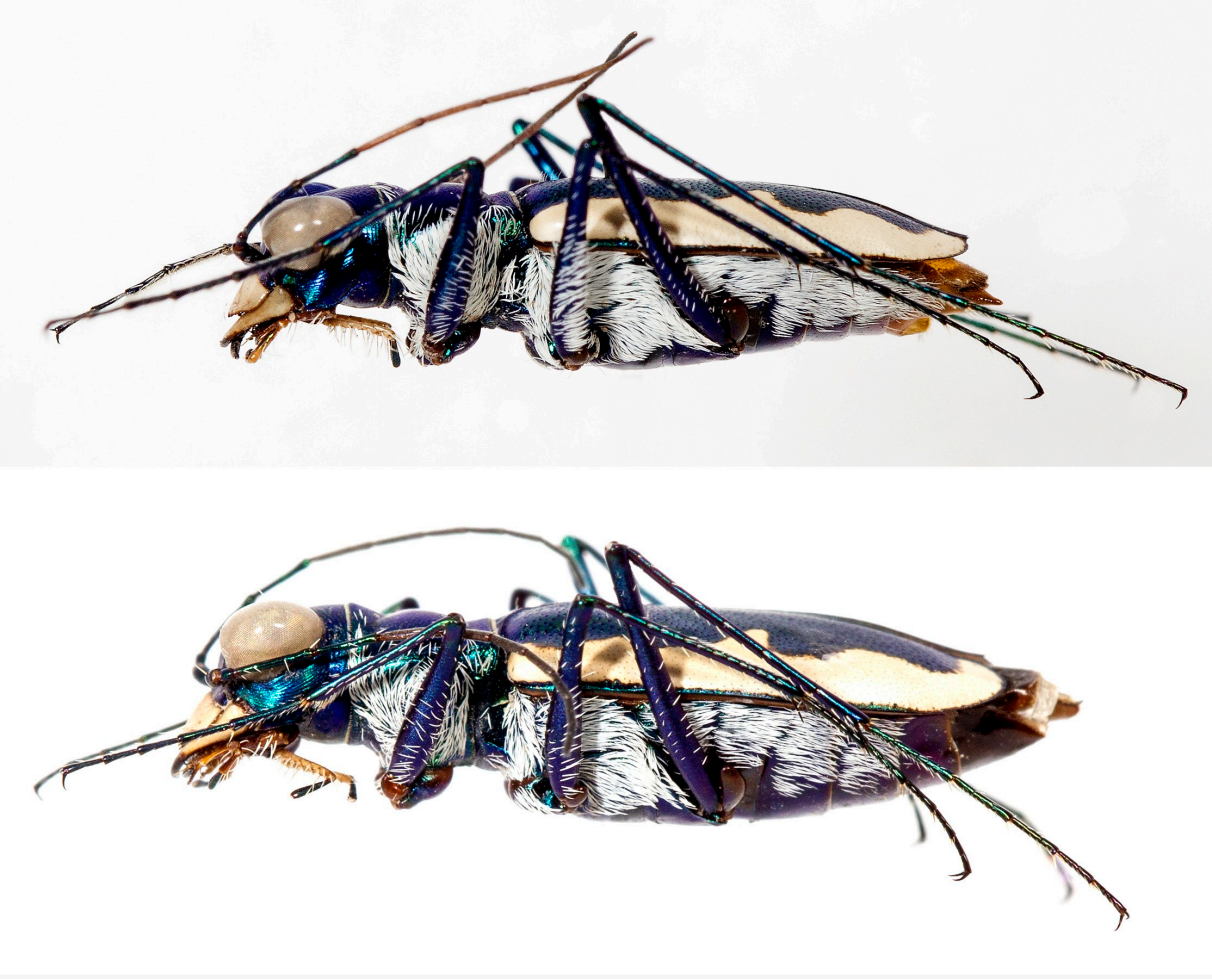
Lateral habitus of *E*. *mecocheila* n. sp. male (top) and *E*. *mecocheila* n. sp. female (bottom).

**Fig 6:**
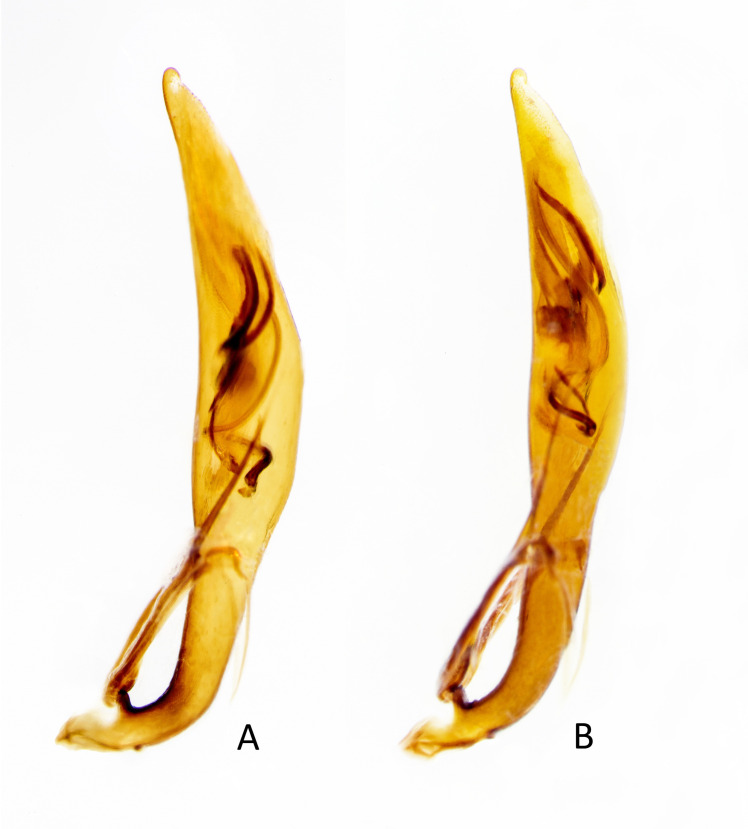
Aedeagus of A) *E*. *mecocheila* n. sp. male, B) *E*. *circumpicta johnsonii* male.

Figs 3, 4A, 4B, 5 and 6A

#### Type material

HOLOTYPE: 1m#, 2.2 mi E Los Rodriguez / June 15, 1990 / Collector W.N. Johnson (USNM). PARATYPES: 4m#, 5f#, 2.2 mi E Los Rodriguez / June 15, 1990 / Collector W.N. Johnson (WNJC). 2m#, 2f#, 2.2 mi E Los Rodriguez / June 15, 1990 / Collector S.J. Roman (SJRC). 1m#, 2 mi S. Las Hermanas, Rio Salado / 17-VII-1991 / Collector J.A. Shetterly (JASC). 1m#, 2 mi S. Las Hermanas, Rio Salado / 24-VII-1988 / Collector R.L. Huber (RLHC). All type specimens labelled: HOLOTYPE or PARATYPE, respectively.

#### Diagnosis

*Eunota mecocheila* **n. sp.** (Figs 3, 4A, 4B, 5 and 6A) can be distinguished from all other *Eunota* by the following combination of characters. This species has a labrum that is more than half as long as it is wide (0.55–0.70, mean = 0.60) with a rounded convex shape (Fig 4A and 4B), body size is 10.2–12.0 mm, mean = 11.0, and lacks setae on the frons, genae and clypeus. The only species that could be confused with *E*. *mecocheila* **n. sp.** are *E*. *circumpicta* and *E*. *togata*. *Eunota circumpicta* maculations are similar to *E*. *mecocheila* **n. sp**. (especially *E*. *c*. *johnsonii*) but *E*. *circumpicta* has a shorter labrum (0.35–0.50) with a less convex shape (Fig 4C and 4D). In addition, *E*. *circumpicta* are larger at 12.3–15.7 mm, mean = 13.1. *Eunota togata* possesses dense decumbent white setae on the frons, genae and clypeus, unlike *E*. *mecocheila* which lacks setae in those areas.

#### Description

Small-sized *Eunota*. Body (Figs 3 and 5) length 10.2–12.0 mm, mean f# 11.4 mm, mean m# 10.6 mm. Head (Fig 4A and 4B) noticeably wider than pronotum due to large eyes, width 2.8–3.5 mm, mean f# 3.3 mm, mean m# 2.9 mm, head variable in color, typically blue-green, similarly colored throughout and with color similar to the pronotum; all head portions glabrous except for two supraorbital setae next to each eye. Frons slightly convex in median area, clearly delimited from clypeus, gradually blending into vertex. Frons surface with distinct longitudinal striae especially in lateral areas bordering eyes, vermiculate-striate in median area. Genae bright polished with deep longitudinal striae abruptly ending at border of vertex. Clypeus irregularly wrinkled to finely vermiculate. Labrum typically with 6–8 setae, ochre-yellow to pale yellow with thin dark brown to black border; male labrum weakly tridentate, somewhat convex, length 0.7–1.1 mm, width 1.3–1.6 mm; female labrum weakly tridentate or unidentate in some specimens, somewhat convex, length 0.8–1.1 mm, width 1.4–1.9 mm. Mandibles medium-sized, ochraceous, dark testaceous along edges. Maxillary palpi mostly yellow with metallic reflections, apical segment dark shiny metallic green to purple. Labial palpi ivory to pale yellow, apical segment dark metallic green to violet. Antennae of normal length, reaching humerus to basal third of elytron in female, to middle of elytra in male; scape with a single subapical seta; pedicel lacking any setae; flagellum antennomeres 3‒4 dark metallic and similar in color to rest of head with ring of apical setae and additional sparse setae throughout, antennomeres 5‒11 ochre-brown, dull-textured without metallic reflections and possessing erect setae in apical rings only, covered with fine pubescence throughout.

Pronotum (Fig 3) 1.6–2.4 mm wide, mean f# 2.2 mm, mean m# 2.0 mm, length 2.1–2.6 mm, mean f# 2.5 mm, mean m# 2.2 mm, width to length ratio 0.55–0.70, slightly polished with metallic finish, color variable but matching head; sparse white decumbent setae present along marginal areas of dorsal surface, some individuals with additional white decumbent setae present along anterior and posterior margins; disc finely rugose to vermiculate with thin but distinct median line and strongly impressed anterior and posterior sulci; notopleural sutures clearly defined, not visible from dorsal view; proepisternum (Fig 5) with decumbent white setae densely covering nearly the entire surface; all other ventral segments of thorax dark testaceous with metallic reflections, lateral areas covered in setae, median areas glabrous.

Elytra (Fig 3) elongate, 6.6–7.8 mm length, mean f# 7.5 mm, mean m# 6.9 mm, shape similar in both sexes, but slightly wider in female, especially toward apical third; sutural spine small, fine microserrations present on elytral apices; elytra color variable, typically green-blue, usually similar but not identical to color of head and pronotum; elytra slightly polished with dense punctures. Subsutural foveae present, but indistinct due to the background punctate texture; elytral maculations present (Figs 3 and 5), with a complete marginal band, a humeral lunule, rounded partial middle band, and apical maculation.

Procoxae and mesocoxae dark testaceous with metallic blue to violet reflections, covered in dense setae; metacoxae dark testaceous with metallic green to violet reflections, nearly glabrous, possessing only a few setae along lateral margins; pro- and mesotrochanters with a single subapical seta, metatrochanters glabrous; femora metallic, with color similar to that of head and pronotum, femoral surface with rows of erect white setae dorsally and ventrally; tibiae mostly colored similarly to femora, clothed with white setae that are sparser and shorter than those of the femora; tarsi colored similarly to the tibiae, first three dilated protarsomeres in male with dense greyish-white setal pads.

Abdominal ventrites 1‒6 dark testaceous with most surfaces covered by metallic reflections; dense white decumbent setae present mostly along lateral third of each ventrite, except ventrite 6 in male and ventrites 5 and 6 in female, which are nearly glabrous. Aedeagus (Fig 6A) shares similarities with *E*. *circumpicta* (Fig 6B), possessing an elongate helical flagellum [21]. The middle/basal third of the *E*. *mecocheila* **n. sp.** aedeagus appears to have a slightly more robust bulge.

#### Etymology

*Eunota mecocheila* **n. sp.** is named for its elongated labrum, derived from Greek: *meco*- = long, -*cheila* = lip.

#### Distribution and habitat

*Eunota mecocheila* **n. sp.** is currently known only from two localities in the northern Mexican state of Coahuila. Beetles were collected in saline muddy ditches. The species has been observed from mid-June to mid-July, and based on the life histories of other closely related *Eunota* [12], it’s adult activity period is likely significantly longer.

## Discussion

This new *Eunota* is closely related to *E*. *circumpicta* (Fig 1) but is separable based on labral proportions and shape (Fig 2), average body size, geographic range and genetic differentiation. The average genetic distance between *E*. *mecocheila* **n. sp.** and *E*. *circumpicta* is 3.3% This is greater than the genetic distance between *E*. *fulgoris* and *E*. *praetextata* (1.4% divergence) and between most *Dromochorus* species [5].

The localities where this new species was collected lie within the Coahuila Ridge and Basin Province of northeastern Mexico [22]. This province has a distinctive physiography, being characterized by elongate ridges separated by broad valleys. The province is located on the eastern side of the North American Cordillera orogenic belt, a north-south belt of high relief, which extends from Alaska south into Central America and includes the Rocky Mountains of North America and the Sierra Madre of Mexico [23]. The province broadly separates the coastal plain of the Gulf of Mexico from the more mountainous terranes of axis of the Cordillera. The province is underlain by Cretaceous and Jurassic marine sediments [24], which were uplifted and folded during Paleogene times by the Laramide orogeny [23], and it is these folds that define much of the present-day ridge (anticlines) and basin (broad synclines) physiography. Many of the anticlines have their cores eroded out, generating secondary basins within the folds themselves (known as “protreros”). This combination of inter- and intra-ridge basins manifests itself as a constellation of basins, which have likely existed for several tens of millions of years. Although most of these basins are not completely landlocked, their rather restricted geometries, combined with the arid climate of the region, results in the formation of saline playa-like environments, habitats that are preferred by members of the genus *Eunota*. In addition, because much of the Mesozoic substrate is of marine origin, there is ample supply of sulfate and calcium to groundwaters, resulting in the formation of gypsum-rich soils in the basins [25]. This unique combination of physiography, climate, and bedrock chemistry likely explains the high level of endemism in the region, including apparently *E*. *mecocheila* n. sp.

We note that this ridge and basin physiography continues north into west Texas in the Big Bend region. No records of the closely related *E*. *circumpicta* or any other *Eunota* have been found in this area. Based on biogeography, it is possible that *E*. *mecocheila* will be found in saline areas of the region. Future efforts should be made to survey for this rare species in west Texas.

## Supporting information

S1 Table Morphological measurements of type material of *E*. *mecocheila* n. sp. (XLS)
